# Herpetology in the 21st Century

**DOI:** 10.3390/ani16060886

**Published:** 2026-03-12

**Authors:** Marc Girondot

**Affiliations:** Laboratoire Ecologie, Société, Evolution, Université Paris-Saclay, Centre National de la Recherche Scientifique et AgroParisTech, 91190 Gif-sur-Yvette, France; marc.girondot@universite-paris-saclay.fr

It is with great honour that I accepted the proposal to become Section Editor-in-Chief of the Herpetology section of the journal *Animals*. First, I would like to sincerely thank all those who have already contributed to making the Herpetology section of *Animals* a recognized reference in the field, foremost among them the previous Section Editor-in-Chief Dr. Franco Andreone, who led this section to a level of excellence that I am committed to maintaining. All Section Board Members deserve to be included in these acknowledgments, as their work is essential to sustaining the journal’s high standards. I also want to thank the reviewers and authors who, day after day, contribute to maintaining the journal’s quality at the highest level.

I have already published several times in the Herpetology section of *Animals*, as the interdisciplinary nature of the journal seemed to align with the evolution of modern science. Of course, more specialized journals—whether focused on particular themes or taxonomic groups—remain valuable, but major advances will come from combining data with other biological models and across different research themes. Indeed, in *Animals*, one can find information on a wide range of taxonomic groups as well as diverse functions and traits, which makes the journal particularly stimulating. The journal’s open access model also appears especially relevant, as it allows all readers to access publications freely. Naturally, publication costs are borne by authors, but the model used by this journal, in line with the practices of its publisher MDPI, is particularly original: by investing time in promoting the journal’s quality through peer review, reviewers receive vouchers that can cover at least part of the publication fees. It is an innovative model that I find highly relevant.

Thus, here I am as Section Editor-in-Chief of the Herpetology section—an appointment that carries a certain irony. As a university professor teaching evolution and phylogenetic taxonomy, I often point out that herpetology, depending on how it is defined, represents a paraphyletic or polyphyletic grouping, which runs counter to the principles I teach. However, as I also teach ecology, I regularly remind students that while phylogenetic taxonomy is indispensable for understanding evolution, selective pressures must also be analyzed within a functional framework—and this is precisely where herpetology finds its full meaning. Phylogenetic constraints and functional constraints are the two pillars that allow us to understand modern biology.

Herpetology is an ancient science. The term itself dates back to 1755 [1], but herpetology as the science of amphibians and reptiles dates from the late 18th century [2]. Herpetology has now fully entered the era of Big Data. Big Data encompasses not only exceptional genomic datasets and functional trait databases, but also environmental data that provide insights unimaginable just a few years ago. This avalanche of data also requires new analytical methodologies—an issue to which I am particularly attentive. The use of statistical approaches capable of handling large datasets has become crucial, even indispensable, to avoid publishing false positives. I am currently preparing a “statistical survival guide” for reviewers. It is essential that articles published in *Animals* remain valid not only today, but also in 10 or even 100 years.

Another field that I believe should be further developed within the Herpetology section of *Animals* is paleontology. The contribution of fossils and fossil faunas is extremely important, for example in improving character polarization in phylogenetic analyses, while keeping in mind that a fossil is never the ancestor itself. Paleontology also helps us understand the functional pressures acting on the characters under study. Another theme I would like to promote is multidisciplinarity—bringing together, for example, chemistry and herpetology, environmental physics and herpetology, but also analyses that couple organisms traditionally included in herpetology with those usually excluded, such as birds or mammals. I would like the Herpetology section of *Animals* to become the leading reference for herpetological studies that are open—open to new data, new methodologies, and also to other taxonomic groups that herpetology can enrich.

This position as Section Editor-in-Chief of the Herpetology section of the journal *Animals* is also an opportunity for me to acknowledge the contributions of two outstanding researchers who played a major role in my academic training. I completed my PhD under the supervision of Dr. Claude Pieau, who discovered the phenomenon of temperature-dependent sex determination in chelonians in 1971 and made exceptional contributions to this field. My habilitation sponsor was Prof. Armand de Ricqlès, an internationally recognized paleontologist renowned for his contributions to the study of thermal physiology in dinosaurs.

Finally, I would like to thank the MDPI editorial team, who greatly facilitate our work as editors, reviewers, and authors, and without whom the section would be far less effective.

## References

[1] Klein J.T. (1755). Tentamen Herpetologiae.

[2] Bonnaterre P.J. (1789). Tableau Encyclopédique et Méthodique des Trois Règnes de la Nature. Erpétologie.

